# Low-photon-number optical switch and AND/OR logic gates based on quantum dot-bimodal cavity coupling system

**DOI:** 10.1038/srep19001

**Published:** 2016-01-11

**Authors:** Shen Ma, Han Ye, Zhong-Yuan Yu, Wen Zhang, Yi-Wei Peng, Xiang Cheng, Yu-Min Liu

**Affiliations:** 1 State Key Laboratory of Information Photonics and Optical Communications, Beijing University of Posts and Telecommunications, Beijing 100876, China

## Abstract

We propose a new scheme based on quantum dot-bimodal cavity coupling system to realize all-optical switch and logic gates in low-photon-number regime. Suppression of mode transmission due to the destructive interference effect is theoretically demonstrated by driving the cavity with two orthogonally polarized pulsed lasers at certain pulse delay. The transmitted mode can be selected by designing laser pulse sequence. The optical switch with high on-off ratio emerges when considering one driving laser as the control. Moreover, the AND/OR logic gates based on photon polarization are achieved by cascading the coupling system. Both proposed optical switch and logic gates work well in ultra-low energy magnitude. Our work may enable various applications of all-optical computing and quantum information processing.

Quantum dots (QDs) in photonic crystal cavity have drawn many attentions for their distinguished potential in implementations and applications of quantum devices and quantum information processing^1^. This QD-cavity coupling system has been studied extensively in the framework of cavity quantum electrodynamic dynamics (CQED) for building blocks of attainable quantum communication and quantum information network in last decade^2^,^3^. The Jaynes-Cummings (JC) model, which describes a two-level atom inside a cavity, plays an important role in quantum optics^4^. Taking the advantage of the long coherence time, numbers of fascinating schemes have been proposed to implement the scalable quantum computation using photonic qubits^5^,^6^,^7^, the deterministic photonic spatial-polarization hyper-CNOT gate^8^, and the quantum gates based on hybrid photon-matter systems^9^,^10^,^11^,^12^.

Recently, several schemes of the all-optical switch in low-photon-number (single photon) regime have been proposed and experimentally demonstated^13^,^14^,^15^. W.L. Chen *et al.* realized an optical transistor in which single stored gate photon was used to control the resonator transmission of source photons^13^. T. Volz *et al.* reported an all-optical switch of single photons in a strongly coupled QD-cavity system, in which the overall switching time was about 50 ps^14^. R. Bose *et al.* demonstrated the nonlinear optical switching between two laser pulses by utilizing the strong coupling between a single QD and an L3 photonic crystal cavity^15^. The temporal switching response was measured as fast as 120 ps while 3 dB and 10 dB contrast were achieved at about 140 and 440 driving photons respectively. Still, higher on-off ratio and speed of all-optical switch is highly desirable. More importantly, to best of our knowledge, the complicated all-optical AND/OR logic gates in low-photon-number regime have not been demonstrated yet. In our work, we investigate the coupling system consisting of a bimodal photonic cavity and a two-level QD in CQED framework. Two pulsed lasers with controllable polarization are adopted to drive the cavity modes. Pulses of both driving lasers are Gaussian shape and a time delay is applied between two lasers. This platform provides us many abundant phenomena and interesting properties. It exhibits an optical switch effect by adjusting the suitable time delay between signal and control laser pulses. The overall switching time is 10 ps order of magnitude in theoretical calculation and meanwhile more than 12.5 dB on-off ratio can be achieved within proper range of coupling strength between QD and cavity. Then we propose a feasible scheme of AND/OR optical logic gates by cascading the QD-bimodal cavity system.

## Results

**Figure 1:**
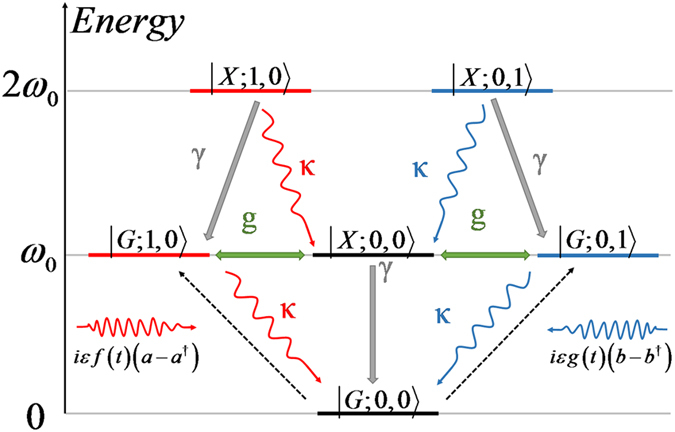
The partial energy structure of QD-cavity coupling system in bare state picture. Here top six energy states are drawn and all possible paths of these six states are marked by their own collapsing rates which show a cavity or a QD transition. Particularly, *g* represents the coupling between cavity modes and QD.

The energy-level structure of the QD-bimodal cavity in bare state picture is illustrated in Fig. 1. Only few pump photons coupled to the cavity mode is assumed due to the low driving energy. State 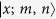 denotes the number states where the value of *x* is *G* (ground state) or *X* (excited state). Letter *m* and *n* represents the photon number states of cavity mode *a* and mode *b* respectively. Two cavity modes are orthogonally polarized at identical frequency and are resonant to the QD. For convenience, we define that laser A has the same polarization as the cavity mode *a* while laser B has the same polarization as the cavity mode *b*. In this scheme, two possible transition paths exist for 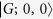 to 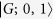 or 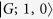. For example, laser B can drive the system directly into state 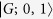 from basic state 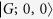 while laser A can drive the system into state 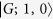 and then transits to state 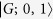 through 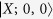 induced by the coupling between QD and both cavity modes. Probability amplitude of state 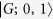 may reduce greatly for the effect called destructive interference. However, when two driving laser pulses arrive simultaneously, the independent transitions in direct and indirect exciting paths can not achieve destructive interference because the transition from state 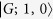 to state 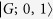 needs an extra period of time.

**Figure 2:**
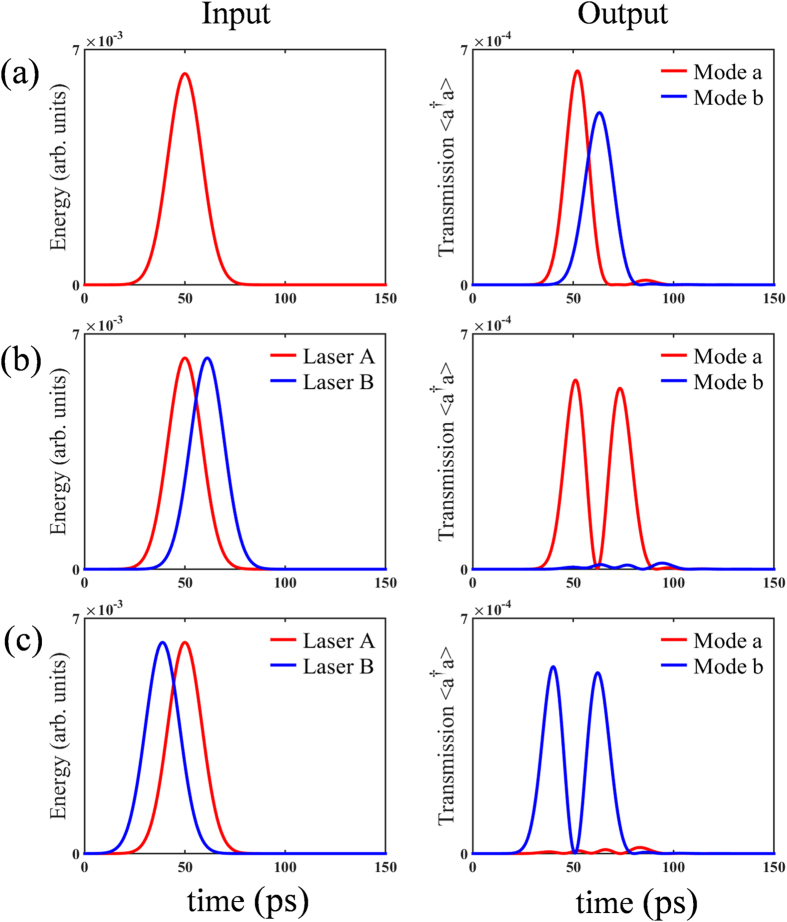
Sequence diagram of the driving lasers and the responsive transmissions. In this figure, we choose these parameters: 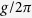 = 20 GHz, Δ*t* = 11.2 ps. (**a**) The sequence diagram of applying single laser A. (**b**) The sequence diagram of applying laser A and laser B with a carefully chosen time delay. (**c**) An opposite situation to (**b**).

Using the Hamiltonian of QD-bimodal cavity coupling system and solving the transient master equation numerically, we obtain the time diagrams of photon transmission by Monte-Carlo method. Figure 2(a) exhibits the fact that transmission of both modes can be expected from the bimodal cavity when the system is driven by single pulsed laser. When two orthogonal driving laser A and laser B with a certain delay (A before B) are adopted to pump the coupling system, only obvious mode *a* photon transmission is observed while mode *b* is almost undetectable as shown in Fig. 2(b). The two peaks of mode *a* transmission derive from the direct transition excited by laser A and the indirect transition excited by latter laser B respectively. The mode *b* transmission is suppressed due to destructive interference induced by indirect transition excited by laser A and direct transition excited by latter laser B. On the contrary, if laser B precedes laser A, mode *a* transmission is suppressed instead as shown in Fig. 2(c). It can be seen that the scheme holds symmetry for the sequence of laser A and laser B.

**Figure 3:**
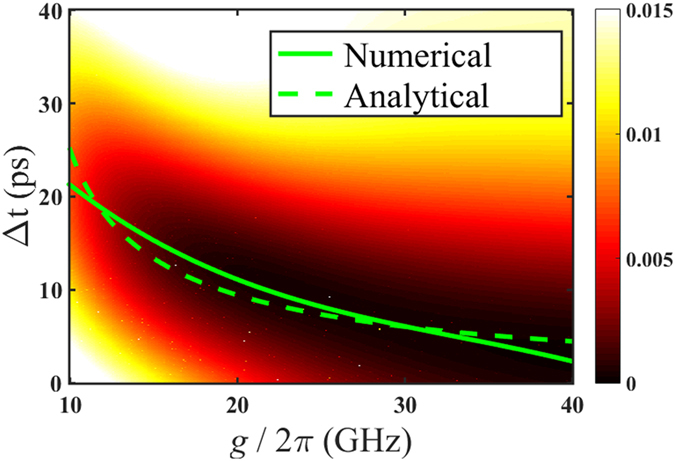
The transmission of QD-cavity system with both driving laser A and B. The color map represents the mode b transmission energy (in arbitrary units) when laser B lags laser A. The abscissa is the coupling strength between QD and cavity. The ordinate is the time delay between the two driving lasers. The solid line denotes the optimal time delay from the numerical scan. The dash line is the curve of equation (1) which is obtained from analytical analysis.

The coupling coefficient between QD and cavity (*g*) and the time delay between pulses of driving laser A and laser B (Δ*t*) are scanned to interpret their influences on single mode transmission. The magnitude of driving lasers are assumed identical. The cavity decay rate and dipole decay rate in numerical simulation are set 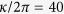
GHz and 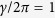
GHz respectively. The color map in Fig. 3 demonstrates the mode *b* transmission energy in the situation laser B lagging behind laser A. When coupling is strong (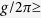
20GHz), mode *b* suppression (only mode A transmission) can occur by choosing proper pulse delay, while the mode *b* cannot be suppressed well in the weak coupling region (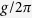 < 20GHz) regardless of Δ*t*. It can be explained by that the transition path in which laser A drives the cavity mode *b* indirectly is not strong enough to achieve destructive interference with the direct transition excited by laser B when the coupling between QD and cavity is weak. For specific *g*, we can determine the optimal Δ*t* which ensures the mode *b* transmission energy minimum. The optimal time delay as a function of coupling coefficient is plotted as solid line in Fig. 3. This function can be fitted by a third order polynomial relation: 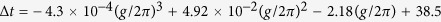, here the unit of Δ*t* is ps and unit of *g* is GHz. The relative ratio between transmission energy of mode *a* and mode *b* reaches about 14.5 dB at 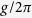 = 20 GHz.

On the other hand, the analytical analysis is adopted to obtain the transition time interval in the QD-bimodal cavity coupling system. Identical non-homogeneous responses of the two driving lasers are assumed. For simplicity, we regard the influence of the driving lasers as an initial state to the system and then solve the motion equation. The Initial state is set 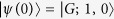 indicating that laser A has been pumped into the cavity. The evolution of the state 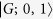 is focused. The time of this state’s probability amplitude from zero to maximum can be approximately expressed as below (more details can be found in the method part):


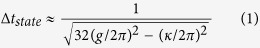


The 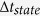 represents the time consumption of transition from state 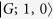 to 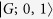. According to equation (1), the time interval is simply related to the coupling strength and cavity decay rate. Quantitatively, it is more sensitive to the coupling strength within the common range^16^,^17^ 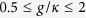. The equation (1) is plotted as dashed line in Fig. 3, which is consistent with the previous numerically obtained optimal pulse delay when the coupling strength is large enough. It proves that the mechanism of proposed scheme for suppression of mode transmission is based on destructive interference effect in QD-bimodal cavity system driven by two laser pulses with delay.

**Figure 4:**
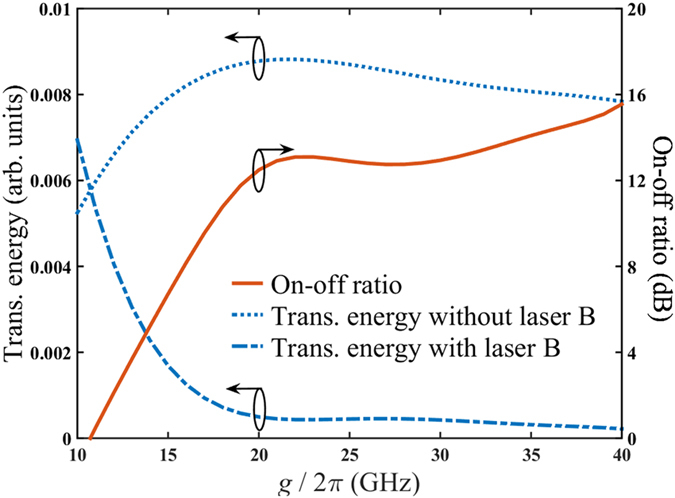
Mode b transmission energy of optical switch with/without laser B pump. The relation between mode b transmission energy and coupling strength is presented. The dotted line denotes the case without driving laser B (single laser A pulse). The dot-dashed line denotes the case with driving laser B (laser B lagging behind laser A). The solid line shows the on-off ratio in dB.

Above scheme can fulfill an all-optical switch in the low-photon-number regime. We regard laser A as input signal and laser B as input control. Meanwhile, the mode *b* transmission is taken as the output signal. Figure 2(a,b) clearly shows how the optical switch works. The presence of the control determines the presence of the mode *b* transmission. In other words, we can “shut down” the output single by applying a control pump with a carefully selected time delay. The relations between mode *b* transmission energy and coupling strength in two cases, with or without driving laser B, are demonstrated in Fig. 4. The time delay between driving lasers follows the numerically obtained optimal time delay. The on-off ratio of the optical switch keeps higher than 12.5 dB when 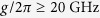. The switching time, defined as time interval between input and output signal, is about 13 ps as illustrated in Fig. 2(a). Here, the responsive transmission of mode *a* photon is useless in this application which may cause energy loss. Moreover, benefiting from the symmetry of proposed scheme to the sequence of driving lasers, it is able to make an artificial choice of the responsive mode transmission which also corresponds to the polarization of photon. We still regard the laser B as a control. The control laser precedes or lags behind the signal laser A determines that the polarization of responsive transmission is same as input signal or orthogonal to signal. It acts like a NOT-gate.

**Figure 5:**
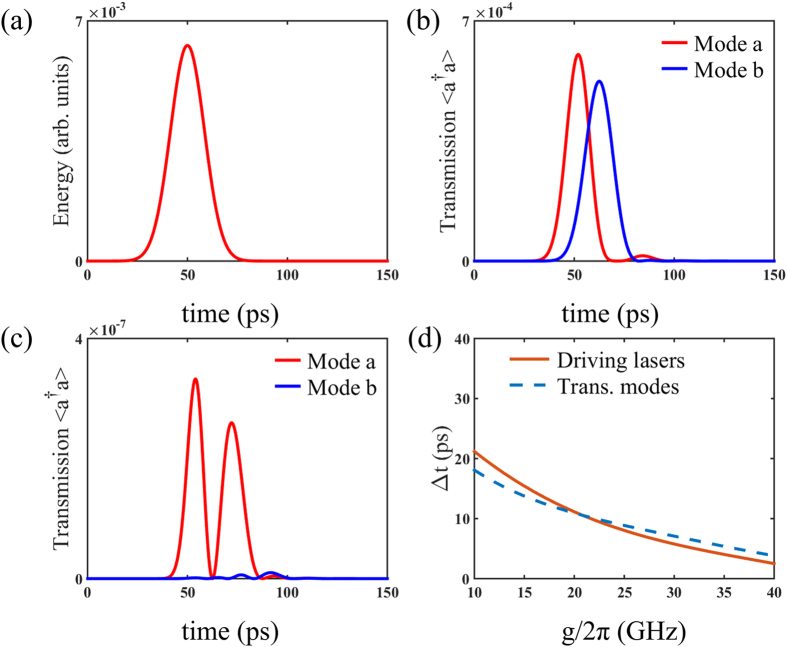
Sequence diagram of the cascaded QD-bimodal cavity system (**a**–**c**) The sequence diagram of the cascaded system. The first subsystem’s transmissions act as the drive to the second subsystem. Suppression of mode b transmission is observed at the output of second subsystem. (**d**) The solid line denotes the optimal time interval between the two driving lasers. The dash line denotes the time interval between the peak of the two transmitted modes.

In the following, we will construct a complex logic gate in the low-photon-number regime based on the switch effect. Two properties of QD-bimodal cavity coupling system make it possible to implement a cascaded system. First, the photon responsive transmission is similar to the Gaussian-shaped pulses. Second, the time interval between mode *a* and mode *b* transmission is similar to the optimal time delay of driving pulses for destructive interference as well. This cascaded system is the foundation to build a logic gate. An exemplary sequence diagram is illustrated in Fig. 5(a–c). We apply single laser A pulse to drive the first QD-cavity subsystem, consequently, both modes transmission will be excited in this system. Mode *b* transmission lags behind the mode *a* as expected. Then we use very low loss waveguides to lead the transmission to the second QD-cavity subsystem as its drive. The suppression of final mode *b* transmission can be observed. Here we set 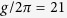
GHz at which the optimal time delay of driving pulses is exactly same as time interval between transmitted modes, as shown in Fig. 5(d). It should be noted that this scheme is symmetric for input laser A and laser B which means the lagged transmitted mode will be shut off in the end.

**Figure 6:**
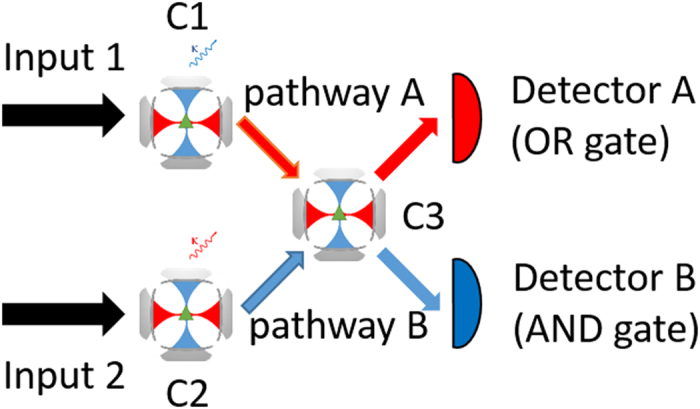
Schematic diagram of the all-optical AND/OR logic gates.

Now we show how to build AND/OR logic gates by cascading the QD-bimodal cavity system as demonstrated in Fig. 6. Three identical QD-cavity subsystems, which are marked as C1, C2 and C3 respectively, are needed. Laser A (B) and mode *a* (*b*) represents *x* (*y*) polarized photon, which are marked as signal R (B). The waveguide between C1 and C3 supports only mode *a* transmission while the waveguide between C2 and C3 supports only mode *b* transmission. The detector A and B are adopted to detect mode *a* photon and mode *b* photon output from C3 respectively. We regard the input signal R as logic 1 and input signal B as logic 0. In the detector side, R and B refers to the same logic as input. N denotes that the detector cannot detect the corresponding photon. For detector A, N is logic 0 while for detector B, N is logic 1.

Three scenarios are discussed in detail:

Scenario I: Both inputs are laser A, then the mode *a* transmission from C1 and the mode *b* transmission from C2 will pump into and drive C3 through the respective waveguide. According to previous analysis, in C3, peak of mode *b* driving lags behind mode *a*, therefore, the mode *b* transmission of C3 will be suppressed. As the results, the detector A can detect photons, while there is almost undetectable photon on detector B. In this scenario, the input and output signals are 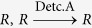; 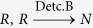. The corresponding logics are 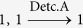; 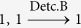.

Scenario II: Both inputs are laser B, opposite to Scenario I, the peak of mode *a* transmission from C1 lags behind the peak of mode *b* transmission from C2. As the results, the detector B can detect photons, while there is almost undetectable photon on detector A. In this scenario: the input and output signals are 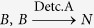; 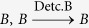. The corresponding logics are 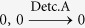; 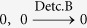.

Scenario III: input 1 is laser A and input 2 is laser B, the peak of mode *a* transmission from C1 and the peak of mode *b* transmission from C2 completely coincides, so photons can be detected both on the detector A and B. The symmetric input scenario holds as well. In this scenario: the input and output signals are 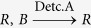; 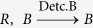. The corresponding logics are 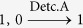; 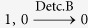.

Straightforwardly, we summarize the scenarios discussed above in Table 1. It can be seen clearly that the detector A plays the role of OR gate, and detector B serves as the AND gate.

**Figure 7:**
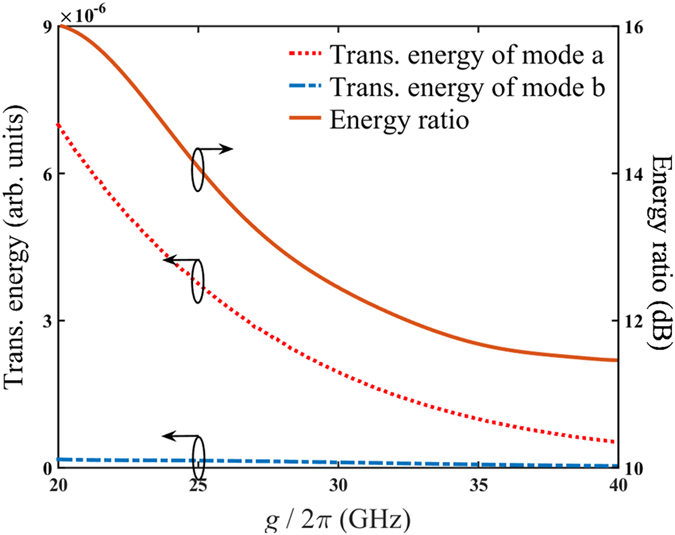
Relation between transmission energy through logic gates and coupling strength of cavities. The dotted line and dash-dotted line denote transmission energy detected by detector A and detector B respectively. The solid line denotes the energy ratio between two detectors. In this simulation, both inputs are set laser A.

Finally, the performance of the AND/OR logic gates is numerically simulated. The relation between transmission energy detected and coupling strength is illustrated in Fig. 7. For simplicity, identical parameters of all three cavities are assumed. We consider the case that both inputs are laser A. Consequently, the mode *b* transmission is expected to be shut at C3. It can be seen that both mode *a* and mode *b* transmission energies decrease with increasing coupling strength. Within the range of 20 GHz
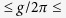
40 GHz, the relative ratio between transmission energy detected by two detectors keeps higher than 11 dB, while the maximum value is about 16 dB at 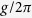 = 20 GHz. Similar phenomenon also holds when inputs are laser B, merely the mode *a* transmission is shut at C3 instead. Thus, the proposed AND/OR logic gates can work well within a relatively large range of coupling strength.

## Conclusion

In summary, we demonstrate a new scheme based on coupling system consisting of QD and bimodal cavity to realize all-optical switch and AND/OR logic gates in low-photon-number regime. When driving the cavity with two orthogonally polarized pulsed lasers at certain pulse delay, suppression of the mode whose polarization is same as latter driving pulse can be observed due to effect of destructive interference. The transmitted mode can be selected by designing the driving laser pulse sequence. When considering one laser as the control, the optical switch effect with at least 12.5 dB on-off ratio is achieved. Moreover, we propose a design of AND/OR logic gates based on photon polarization. This is fulfilled by cascading the QD-cavity system. The proposed scheme shows a feasibility of applications in quantum information processing, especially in optical quantum computing.

## Method

In this work, we consider the QD-bimodal cavity coupling system in framework of CQED. It is assumed that two modes of the cavity are orthogonally polarized at identical frequency and are resonant to the QD. The Hamiltonian of the coupling system is described by


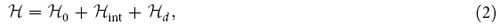


where


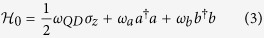



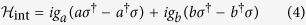



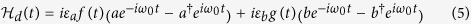


Here, 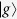 and 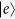 denote the ground state and the excited state of the QD respectively. 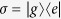 is the QD’s lowering operator. 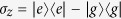 is the QD’s Pauli operator. *a* and *b* are the annihilation operators of the two cavity modes with frequency 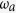 and 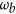. We switch the Hamiltonian above to a frame rotating with the laser frequency 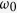. The new Hamiltonian satisfies the conversion 
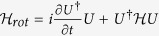
 where 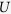 is given by 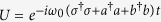. The new Hamiltonian becomes:


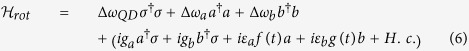


where 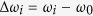 (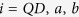) are the detuning of QD, cavity mode *a* and cavity mode *b*. 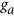 and 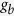 describe the coupling strengths between the QD and cavity modes. We consider the driving Hamiltonian 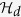 which describes two pulsed lasers with controllable polarization capable of driving both cavity modes respectively and directly. 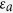, 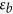 are the exciting strengths and 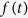, 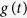 represent the shapes of the applied laser fields. 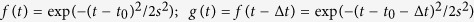. Here, 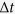
is the time delay between pulses and 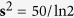 ps. The driving lasers are Gaussian pulses which have the same shape and the same energy (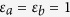
GHz). We assume the photons of lasers are resonant to the cavity modes respectively (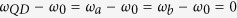). This assumption can be achieved by using electrical field manipulation or controlling temperature^18^,^19^. The coupling strengths between the QD and cavity modes can be equal (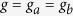) when the magnitudes of mode electromagnetic field at the location of QD are equal and the polarization angles between the QD dipole and both modes are equal at the same time^20^. The Hamiltonian under the rotating frame with the above assumptions will be simplified as


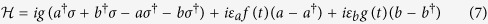


The dissipative dynamics is described by the master equation


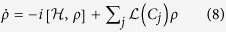


Here 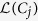 is Lindblad superoperator with the definition of 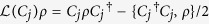. The loss channel of each cavity and QD transition will be represented by the collapsing operator 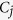, where 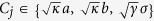. We perform the analysis with these achievable decay rates: the cavity decay rate is 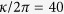  GHz and the dipole decay rate is 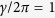  GHz. The master equation is numerically solved by Monte-Carlo method.

On the other hand, the analytical analysis is adopted to obtain the transition time interval in the QD-bimodal cavity coupling system. Identical non-homogeneous responses of the two driving lasers are assumed. The non-homogeneous differential equations are not solved strictly. We regard the influence of the driving lasers as an initial state to the system. We proceed to solve the motion equation from expanding the state vector into a linear combination of the bare states: 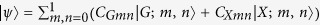. The evolution satisfies the Schrödinger equation, i.e. 
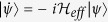
. We assume an initial state instead of considering the driving terms to avoid a series of complicated calculations. Then, effective Hamiltonian is given by 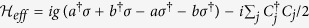. We only care about the possibility amplitude of state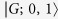:


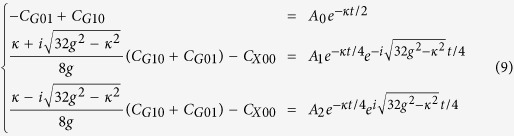


The C_xmn_ on the left side of above equations are the none zero expansion of dressed states corresponding to the Eigen energy on the right side in the bare state representation. Strictly, The 
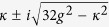
 in Eigen energies of the dressed states should be 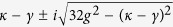. Here 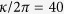 GHz, 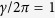 GHz, we therefore ignore the influence of 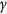. Considering the initial state 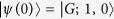, we get the following solution:


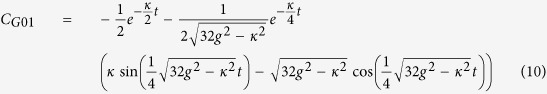


Then, the probability of state 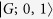 is given by 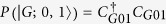. Similar to the Rabi oscillations, transition of the system in state 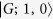 to state 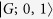 will spend a period of time. The period of probability 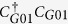 from zero to maximum can be simply approximated by:


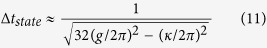


## Additional Information

**How to cite this article**: Ma, S. *et al.* Low-photon-number optical switch and AND/OR logic gates based on quantum dot-bimodal cavity coupling system. *Sci. Rep.* **6**, 19001; doi: 10.1038/srep19001 (2016).

## Figures and Tables

**Table 1:** Logic table of schematic AND/OR logic gates based on photon polarization.

Input 1	Input 2	C3 pulse sequence	Detector A (OR)	Detector B (AND)
R (1)	R (1)	R B	R (1)	N (1)
B (0)	B (0)	B R	N (0)	B (0)
R (1)	B (0)	R	R (1)	B (0)
		B		
B (0)	R (1)	B	R (1)	B (0)
		R		

## References

[b1] Wei H. R. & Deng F. G. Universal quantum gates on electron-spin qubits with quantum dots inside single-side optical microcavities. Opt. Express 22, 593–607 (2014). 24515020 10.1364/OE.22.000593

[b2] Kimble H. J. The quantum internet. Nature (London) 453, 1023–1030 (2008). 18563153 10.1038/nature07127

[b3] Ritter S. *et al.* An elementary quantum network of single atoms in optical cavities. Nature (London) 484, 195–200 (2012). 22498625 10.1038/nature11023

[b4] Walls D. F. & Milburn G. J. Quantum Optics (Springer-Verlag, Berlin-Heidelberg, 2008).

[b5] Wei H. R. & Deng F. G. Scalable photonic quantum computing assisted by quantum-dot spin in double-sided optical microcavity. Opt. Express 21, 17671–17685 (2013). 23938640 10.1364/OE.21.017671

[b6] Wang H. F., Zhu A. D., Zhang S. & Yeon K. H. Optically controlled phase gate and teleportation of a controlled-NOT gate for spin qubits in a quantum-dot-microcavity coupled system. Phys. Rev. A 87, 062337 (2013).

[b7] Li X. *et al.* An all-optical quantum gate in a semiconductor quantum dot. Science 301, 809–811 (2003). 12907794 10.1126/science.1083800

[b8] Ren B. C., Wei H. R. & Deng F. G. Deterministic photonic spatial-polarization hyper-controlled-not gate assisted by quantum dot inside one-side optical microcavity. Laser Phys. Lett. 10, 095202 (2013).

[b9] Hu C. Y. *et al.* Giant optical Faraday rotation induced by a single-electron spin in a quantum dot: Applications to entangling remote spins via a single photon. Phys. Rev. B 78, 085307 (2008).

[b10] Hu C. Y., Munro W. J. & Rarity J. Deterministic photon entangler using a charged quantum dot inside a microcavity. Phys. Rev. B 78, 125318 (2008).

[b11] Bonato C. *et al.* CNOT and Bell-state analysis in the weak-coupling cavity QED regime. Phys. Rev. Lett. 104, 160503 (2010). 20482035 10.1103/PhysRevLett.104.160503

[b12] Wei H. R. & Deng F. G. Universal quantum gates for hybrid systems assisted by quantum dots inside doublesided optical microcavities. Phys. Rev. A 87, 022305 (2013).

[b13] Chen W. L. *et al.* All-Optical Switch and Transistor Gated by One Stored Photon. Science 341, 768 (2013). 23828886 10.1126/science.1238169

[b14] Volz T. *et al.* Ultrafast all-optical switching by single photons. Nature Photon 181, 605–609 (2012).

[b15] Bose R. *et al.* Low-Photon-Number Optical Switching with a Single Quantum Dot Coupled to a Photonic Crystal Cavity. Phys. Rev. L 108, 227402 (2012). 10.1103/PhysRevLett.108.227402 23003653

[b16] Majumdar A. *et al.* Proposed coupling of an electron spin in a semiconductor quantum dot to a nanosize optical cavity. Phys. Rev. L 111, 027402 (2013). 10.1103/PhysRevLett.111.027402 23889441

[b17] Englund D. *et al.* Resonant excitation of a quantum dot strongly coupled to a photonic crystal nanocavity. Phys. Rev. L 104, 073904 (2010). 10.1103/PhysRevLett.104.073904 20366887

[b18] Ren Q. *et al.* Spin-resolved Purcell effect in a quantum dot microcavity system. Nano Lett. 12, 3455 (2012). 22698083 10.1021/nl3008083

[b19] Kistner C. *et al.* Demonstration of strong coupling via electro-optical tuning in high-quality QD-micropillar systems. Opt. Express 16, 15006 (2008). 18795037 10.1364/oe.16.015006

[b20] Srinivasan K. & Painter O. Mode coupling and cavity-quantum-dot interactions in a fiber-coupled microdisk cavity. Phys. Rev. A 75, 023814 (2007).

